# Compartmental changes in the body-fluid contributions to the plasma volume restoration during recovery from dehydration following heat acclimation

**DOI:** 10.1186/2046-7648-4-S1-A108

**Published:** 2015-09-14

**Authors:** Mark J Patterson, Jodie M Stocks, Nigel AS Taylor

**Affiliations:** 1 Centre for Human and Applied Physiology, School of Medicine, University of Wollongong, Wollongong, Australia

## Introduction

Since water moves freely among fluid compartments, it was of interest to track whole-body fluid movements during a resting recovery from extended exercise in the heat, but without rehydration. This mechanism was investigated before, during and following an extended heat-acclimation experiment.

## Methods

Eight males were heat acclimated over 17 days (40 °C, 60 % relative humidity) using the controlled hyperthermia technique (deep-body temperature clamped at 38.5 °C). Before (day 1), during (day 8) and after acclimation (day 22), whole-body, inter-compartmental fluid movements were tracked, firstly during progressive dehydration (heat stress test: 30 min seated rest plus 90 min cycling [same posture]: 40 °C) and then during passive recovery (30 min seated rest: 28 °C), all without fluid replacement. Changes in whole-body, intra- and extracellular body-fluid volumes during heat-stress tests and recovery were quantified using combined radionuclide- and dye-dilution techniques. These data illustrate not just compartmental contributions to fluid loss, but fluid movements during the transition from exercise to recovery.

## Results

During exercise in the heat, plasma volume reductions of 9.0 % (SEM 0.9: day 1), 12.4 % (SEM 1.6: day 8) and 13.6 % (SEM 1.2: day 22) were observed, with whole-body fluid losses on days 8 and 22 significantly exceeding day 1 (*P*<0.05). This whole-body fluid loss continued during recovery, as sweating continued for some time, with whole-body sweat losses averaging 229 (day 1), 303 (day 8) and 392 mL (day 22). However, recovery of the plasma volume commenced very quickly, even without fluid being consumed. Indeed, during the 30-min recovery, plasma volume increments of 1.9 % (day 1: 65 mL), 3.2 % (day 8: 112 mL) and 5.4 % (day 22: 180 mL), relative to volumes measured immediately following exercise, were observed. This pattern was consistent with an enhancement of the plasma volume restoration as heat acclimation progressed. Prior to commencing heat acclimation, the relative interstitial contribution dominated fluid loss during recovery, with the intracellular share being about half that of the interstitium. Thus, fluid loss occurred only from the intracellular and interstitial reservoirs, with the former sustaining the interstitial volume and thereby permitting a partial intravascular recovery. The mechanism underlying this outcome appeared to be a significant elevation of the plasma and, presumably also, the interstitial osmolality beyond 90 min of dehydration on days 8 and 22. As a consequence, the intracellular fluid contribution to this plasma recovery gradually became more pronounced. This sustained the interstitial fluid compartment, and thereby permitted a partial intravascular recovery, even before fluid was consumed. Whilst the intracellular fluid reduction during exercise represented <30 % of the total water lost, during recovery, the absolute fluid loss from this compartment more than doubled.

## Discussion

This snapshot of body-fluid dynamics at the end of recovery illustrates how inter-compartmental fluid movements may help defend against post-exercise hypotension, with the intracellular fluid reserves behaving as a temporary reservoir from which the plasma fraction is restored via the interstitium prior to commencing fluid replacement. Moreover, the recovery of the plasma volume following progressive dehydration was more rapid following heat adaptation.

